# Molecular characterization of 20 small supernumerary marker chromosome cases using array comparative genomic hybridization and fluorescence *in situ* hybridization

**DOI:** 10.1038/s41598-017-10466-z

**Published:** 2017-09-04

**Authors:** Mingran Sun, Han Zhang, Guiying Li, Carrie J. Guy, Xianfu Wang, Xianglan Lu, Fangchao Gong, Jiyun Lee, Susan Hassed, Shibo Li

**Affiliations:** 1 Department of Pediatrics, University of Oklahoma Health Sciences Center, Oklahoma City, OK 73104 USA; 2 Department of Hematology and Oncology, Anshan Hospital, the First Hospital of China Medical University, Anshan City, Liaoning, 114000 China; 3 Key Laboratory for Molecular Enzymology and Engineering, College of Life Sciences, Jilin University, Changchun City, Jilin, 130012 China; 4 Center for Reproductive Medicine, Center for Prenatal Diagnosis, the First Hospital of Jilin University, Changchun City, Jilin, 130021 China; 5 Department of Urology, the First Hospital of Jilin University, Changchun City, Jilin, 130000 China; 6 Department of Pathology, College of Medicine, Korea University, Seoul, 02841 Korea

## Abstract

The variability of a small supernumerary marker chromosome (sSMC)-related phenotype is determined by the molecular component, the size, and shape of the marker chromosome. As fluorescence *in situ* hybridization has limitations regarding the resolution, efficiency, and accuracy. Recently, array comparative genomic hybridization (aCGH) was used for sSMC characterization. In this study, twenty cases with sSMCs were characterized by aCGH and FISH. Chromosomal origin of the marker chromosomes were successfully identified in seventeen of them. For the three cases with negative aCGH results, two of them were more likely due to that the sSMCs only contained centromere heterochromatin, whereas the reason for the remaining case with negative aCGH finding was uncertain. In order to establish a stronger genotype-phenotype correlation for clinical service in the future and avoid miss characterization, more sSMC cases were needed to be detailed characterized. This will help to clarify the variable clinical characteristics of sSMCs and provide additional information to aid clinical service and future research.

## Introduction

Small supernumerary marker chromosome (sSMC) is a structurally abnormal chromosome that cannot be clearly characterized by conventional banding cytogenetics alone and is equal in size or smaller than a chromosome 20 of the same metaphase spread^1^. Small supernumerary marker chromosomes occur in 0.072–0.075% of prenatal cases and 0.044% of newborn cases^2, 3^. Approximately 66.7% of sSMC are *de novo* and 30% are clinically abnormal^2^. Currently, a *de novo* sSMC remains a challenge for physicians and genetic counselors regarding the clinical outcomes, except for the few sSMCs which outcomes have been well characterized including: i(5p), i(9p), i(12p), i(18p), der(22)t(11;22), sSMC(15) containing the Prader-Willi Syndrome/Angelman Syndrome (PWS/AS) critical region, and sSMC(22) containing the critical region for cat eye syndrome^4^. In order to predict outcomes of sSMCs, an accurate and detailed characterization of the sSMC combined with genotype-phenotype correlation studies are necessary^1, 5–10^.

Fluorescence *in situ* hybridization (FISH) based methods have been considered as the standard method to detect the origin of sSMC^11–14^. However, this assay has limitations regarding the accuracy, resolution, and efficiency. For the sSMCs with unknown origin, sometimes multiple attempts were needed to figure out the origins. Additionally, FISH may result in partial classification of complex sSMC derived from two different chromosomes if positive FISH result with single probe was obtained and assumed as the only origin of sSMC^15, 16^. Thus, it is inefficient and costly to use FISH to determine the chromosome origin. In 2004, BAC clone array comparative genomic hybridization (aCGH) has been applied to overcome disadvantage of FISH assay^17^. Recently, the oligonucleotide aCGH assay has been developed and it has gradually replaced BAC clone array CGH^10, 18, 19^, became as a sensitive technique for detecting copy number changes at the whole genome level, it not only can detect genomic copy number changes, but also define breakpoints, and the genes involved. With up to millions of probes representing whole genome on one chip, it also has locus or gene specificity. In this study, aCGH successfully identified the chromosome origin of sSMCs in seventeen of twenty cases. Of these, one of the complex sSMCs had very unique components involving chromosome 3 and 11 and had not been previously reported.

## Results

Between 2000 and 2014, a total of twenty cases with sSMC initially were detected by G-binding karyotype. Routine cytogenetic analysis showed mosaic marker chromosome in 6 out of the 20 cases (P1, P7, P9, P13, P14, P19). All the twenty cases were subjected to aCGH assay, and seventeen of them were successfully identified the chromosome origin. The genotype and phenotype of all twenty cases were summarized in Table 1.

**Table 1:** Summary of cytogenetic, aCGH and FISH findings in small supernumerary marker chromosomes.

Case #	Karyotype Result/Mosaicism	*de novo/*Inherited	Array CGH result	FISH	Clinical features/Reason of study
P1	47,XX, +mar[62.5%]/46,XX[37.5%]	N.D.	arr12p13.33p11.21(15,521–31,936,521)x3–4	ish i(12)(p13.33p11.21)(TEL++)	Abnormal diaphragm
P2	47,XX, +mar [100%]	N.D.	arr15p11.1q13.3(18,420,959–30,704,996)x3	ish idic(15)(p11.1q13.3) (CEP15++, GABRB3+)	DD, short stature, seizures, hypotonia in infancy, behavior problems (rage, aggression) and precocious puberty
P3	47,XX, +mar [100%]	N.D.	arr15p11.1q13.3(18,420,959–30,710,269)x3	N.D.	DD, at 17months was developed level of 10 months, head size (97%ile)
P4	47,XX, +mar [100%]	N.D.	arr15 p11.1q13.3(18,252,731–29,624,999)x4	ish inv dup(15)(p11.1q13.3) (D15Z1++, SNRPN++)	DD, slight hypertonia, seizures
P5	47,XX, +mar [100%]	*de novo*	arr15p11.1q11.2(18,420,959–22,930,675)x4 arr15q11.2q13.1(22,938,482–26,208,665)x6 arr15q13.1(26,239,257–26,803,401)x4	ish inv dup(15)(GABRB3++++, D15Z1++)	DD, at 13 months of age, she had microcephaly, multiple hemangiomas, a cafe-au-lait mark, brachydactyly, metopic craniosynostosis, retromicrognathia
P6	47,XX, +mar [100%]	N.D.	arr15p11.1q13.3(18,262,731–29,850,034)x4	ish inv dup(15)(p11.1q13.3) (SNRPN++, D15Z1++)	DD, hypotonia
P7	47,XY, +mar[60%]/46,XY[40%]	maternal	arr16p11.2q12.1 (28,825,250–46,356,412)x3	N.D.	Fetus with sSMC, Mother normal
P8	47,XY, +mar [100%]	N.D.	arr21p11.2q21.1 (9,725,004–15,550,180)x3	ish min(21)(p11.2q21.1) (CEP13/21+)	Syndactyly, scoliosis
P9	47,XY, +mar[43.7%]/46,XY[53.3%]	N.D.	arr21p11.2 q11.2 (9,725,004–13,350,028)x3	ish min(21)(p11.2q21.2) (CEP13/21+)	Choroid plexus cyst, anomalies of skull, mild macrocephaly
P10	47,XX, +mar [100%]	N.D.	arr22q11.1q11.21(14,434,579–17,269,529)x4	ish inv dup(22)(q11.1q11.21) (TUPLE1++)	Multiple congenital anomalies, single umbilical artery, absent right kidney, congenital heart defect, total anomalous pulmonary venous return, preauricular skin tag, hirschsprung disease
P11	47,XX, +mar [100%]	N.D.	arr22q11.1q11.21(17,068,186–18,651,673)x3	ish min(22)(q11.1q11.21) (WCP22+)	DD, aortic arch anomaly, FTT, positional plagiocephaly, dysphagia, microcephaly, imperforate anus, rectovaginal fistula, total anomalous pulmonary venous connection, g-tube, retinal defect, right pre-auricular ear tag, broad nasal bridge, small widely spaced eyes, frontal bossing
P12	47,XY, +mar [100%]	maternal	Fail to detect the chromosome origin	ish der(14 or 22)(CEP14/22+)	Maternal age, Mother normal
P13	47,XY, +mar[90%]/46,XY[10%]	N.D.	Fail to detect the chromosome origin	ish der(14 or 22)(CEP14/22+)	DD,Short stature (−2.93 SD), poor speech, depressed nasal bridge, narrow head, low-set ears, dental crowding
P14	47,XX, +mar[60%]/46,XX[40%]	N.D.	arr3q26.3q29(170,924,999–199,501,827)x3, arr11q23.3q25(118,874,999–134,440,034)x3	ish der(3 or 11) t(3;11)(q26.3qter; q23.3qter) (RP11–362K14+; RP11–496N6+)	DD, seizures, trichotillomania, behavior problems, madelung deformity
P15	47,XY, +mar [100%]	Mother has balanced t(14q;16p)	arr14q11.2(19,694,999–23,534,999)x3, arr16p13.3p13.13(14,999–11,834,999)x3	ish der(14)t(14;16)(q11.2;p13.13) (CEP14+, WCP14+, @16pter+)	DD, bilateral cleft palate, small chin, cutis marmorata, microcephaly, bilateral clubfeet, bilateral inguinal hernia, umbilical hernia, contractures of the finger joints
P16	47,XY, +mar [100%]	Mother has balanced t(11q;22q)	arr11q23.3q25(116,189,388–134,444,816)x3, arr22q11.1q11.21(15,635,833–18,691,906)x3	N.D.	DD, pre-auriclar tag, prominent nose, narrow chest, and small penis, cleft palate, atrial septal defect and patent foramen ovale, hip dysplasia, chronic kidney disease, and possible seizures.
P17	47,XY, +mar [100%]	N.D.	arr11q23.3q25(116,186,822–134,444,816)x3, arr20p12.2(11,232,997–11,870,134)x3, arr22q11.1q11.21(14,434,579–18,691,906)x3	N.D.	DD, partial diaphragmatic hernia, mild tachypnea, malrotation, laryngomalacia, pre-auricular tags, microcephaly, plagiocephaly, high palate, mild micorgnathia, poor head control, overlapping toes
P18	47,XX, + mar [100%]	N.D.	arr18p11.32p11.21(133,843–15,082,862)x4	N.D.	Small low-set ears, patent ductus arteriosus, horseshoe kidney, small head size (6^th^ %ile)
P19	48–50,XX, +mar1[100%], +mar2[100%], +mar3[N.D.], +mar4[N.D.]	*de novo*	arr5p12 q12.1 (43,011,012–61,225,122)x3, arr12p11.1 q12 (33,664,333–41,698,061)x3, arrXp11.22 q12 (51,034,254–64,960,506)x3–4	ish min(X)(p11.21q11.1)(CEPX+) ish min(X)(p11.22q12)(CEPX+) ish min(5)(p12 q12.1)(WCP5+) ish min(12)(p11.1q12)(CEP12+)	Heart was too small to be medical sized, centriculap septal defect, trileaflet aortic valve, left aortic arch, unusual origin of right coronary artery, almost interrupted aortic arch, small transverse arch
P20	47,XX, +mar [100%]	maternal	Fail to detect the chromosome origin	N.D.	Fetus with sSMC, Mother normal

N.D: not done. DD: development delay. FTT: failure to thrive.

Of these seventeen cases with chromosome gain detected by aCGH, two isochromosome sSMCs derived from chromosome 12 (P1) and 18 (P18), respectively. Five originated from chromosome 15 containing the PWS/AS critical region (15q11q13) (P2, P3, P4, P5, P6). Two sSMCs derived from chromosome 21 (P8, P9). Two sSMCs originated from chromosome 22 (P10, P11). Two cases had complex sSMCs (P14, P15), case P14 had a rare recombination between 3q26.3qter and 11q23.3 and had not been previously reported, another complex sSMC (case P15) was previously reported by our laboratory. Two cases had der(22)t(11;22)(q23.3;q11.21) (P16, P17), and in case P17, beside the gain of der(22)t(11;22) syndrome, aCGH analysis revealed another gain of the 20p12.2 region. One case (P19) had multiple marker chromosome, aCGH analysis revealed three gains at different chromosomes, a 13.9 Mb gain of the Xp11.22 q12 region [arrXp11.22 q12 (51,034,254–64,960,506)x3–4], a 18.2 Mb gain of the 5p12q12.1 region [arr5p12q12.1 (43,011,012–61,225,122)x3], and a 8.0 Mb gain of the 12p11.1q12 region [arr12p11.1q12 (33,664,333–41,698,061)x3].

In the remaining three cases (P12, P13, P20) with negative result of aCGH, FISH assay utilizing probe of CEP14/22 revealed five distinct signals on interphase cell in cases P12 and P13, whereas case P20 had not performed FISH for further investigation due to limited specimen amount.

## Discussion

In this study, twenty cases were analyzed by aCGH, and seventeen of them were successfully identified the chromosome origin (Table 1). To our best knowledge, one of the complex sSMCs, involving chromosomes 3 and 11, has not been previously reported.

**Figure 1:**
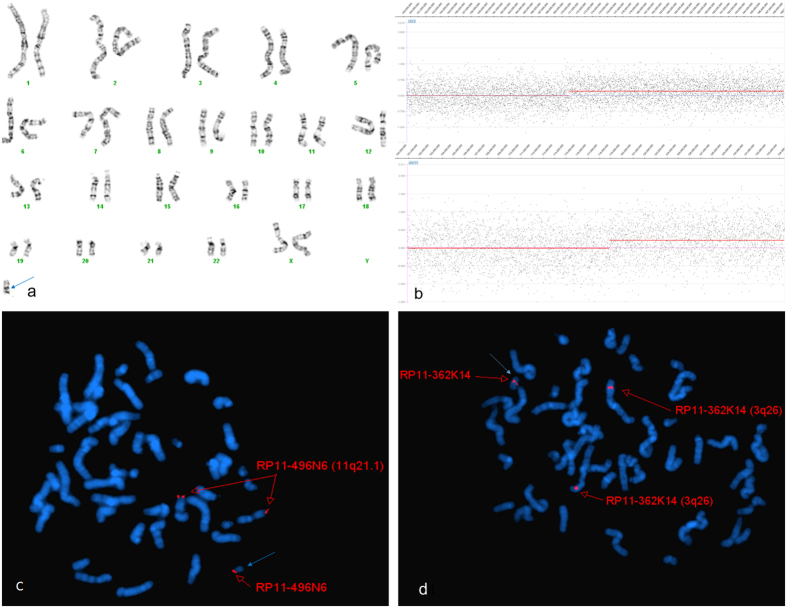
Cytogenetic and molecular results of case P14. The marker is highlighted by a blue arrow in a, d, and e. (**a**) In twelve out of the twenty studied cells, G-banding revealed a karyotype 47,XX, + mar. In the other eight cells, G banding revealed a normal karyotype 46,XX. (**b**) The sSMC of case P14 characterized after aCGH covering 28.6 Mb [arr3q26.3qter(170,924,999–199,501,827)x3] in chromosome 3 and 15.6 Mb [arr11q23.3qter(118,874,999–134,440,034)x3] in chromosome 11. (**c**) and (**d**) Confirmatory FISH results of this sSMC using the RP11-362K14 probe specific for 3q26 and the RP11-496N6 probe specific for 11qter revealed that 82% of the cells had three distinct signals.

Array CGH has many advantages that make it extremely useful for characterizing sSMCs. It clearly and easily determines the components of sSMCs in a single assay. This advantage was especially useful for complex sSMCs and multiple sSMCs. Complex sSMCs is a subgroup of sSMCs which consist of chromosomal materials derived from more than one chromosome^1^. This subgroup may be mischaracterized if only FISH has been used for characterization due to miss another part of sSMC^10, 15, 16^. In this study, both cases P14 (Fig. 1) and P15 were found to have complex sSMCs based on the array CGH findings. Routine chromosome analysis of P14 showed that the sSMC was mosaic in this patient, 60% cells had a small marker chromosome and the 40% cells were normal (Fig. 1a). A subsequent aCGH study revealed that the mosaic sSMC originated from part of the long arm of chromosome 3 (3q26.3qter, 28.9 Mb) and part of the long arm of chromosome 11 (11q23.3qter, 15.6 Mb) (Fig. 1b). Confirmatory FISH using the RP11-362K14 targeting 3q26 and the RP11-496N6 probe targeting 11qter revealed that 82% of the cells had three signals, which is consistent with the aCGH result (Fig. 1c,d). This complex sSMC had a rare recombination and had not been previously reported. Case P14 showed developmental delay, seizures, trichotillomania, behavior problems, and madelung deformity. The sSMC overlapping chromosome 11q23.3qter was rare, one similar case had been documented in sSMC database created by Dr. Thomas Liehr^20^. The genotype of this patient was 47,XY,del(11)(q22), + mar[100%], and the sSMC was inv dup(11)(qte- > q22::q22- > qter). The patient showed mental retardation, developmental delay or structural anomalies detected at birth^20^. In the sSMC database, four patients carrying a sSMC smaller than 3q23.6qter were clinical abnormal indicated that chromosome 3q26.3qter was a dosage sensitive region and might associate with developmental delay, seizures, behavior problems, and dysmorphic features^20^. On the other hand, chromosome 3q29 duplication has been suggested to be associated with mild to moderate mental retardation and minor dysmorphic features (Chromosome 3q29 microduplication syndrome, OMIM 611936). Thus, this complex sSMC der(3 or 11) t(3;11)(qter- > q26.3:: p13.13- > pter) might responsible for the phenotype of P14 and likely lead to developmental delay, seizures, behavior problems, and dysmorphic features. Case P15 was previously reported by our laboratory and it might be associated with non-syndromic Pierre Robin sequence^21^.

**Figure 2:**
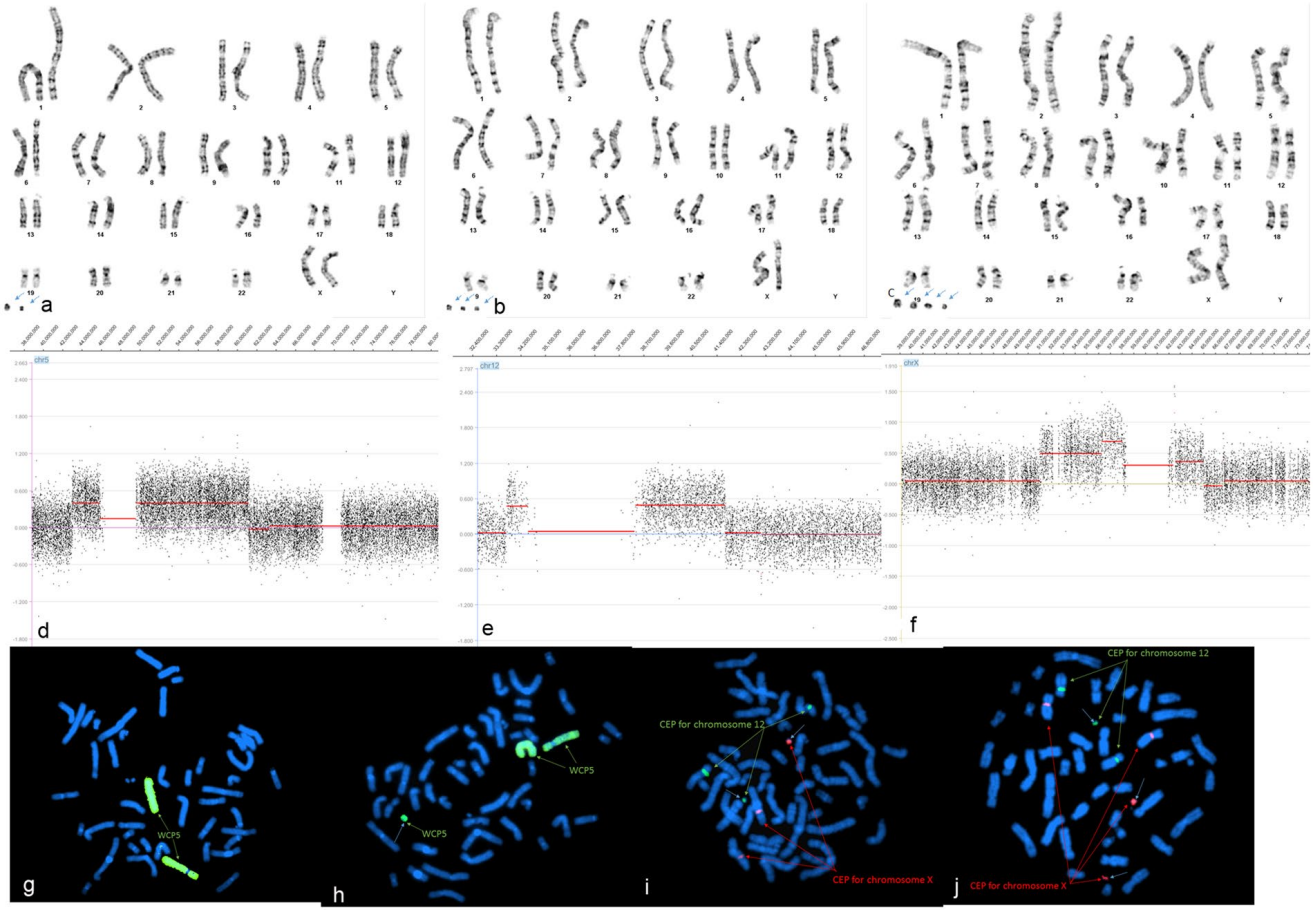
Cytogenetic and molecular results of case P19. The marker is highlighted by a blue arrow in a, b, c, f, i, and j. (**a**,**b**), and (**c**) G-banding revealed a karyotype 48-50,XX, +mar1, +mar2, +mar3, +mar4 in a mosaic form. (**d**) Array CGH detected gains at chromosome 5p12 to 5q12.1. The log2 ratio was approximately 0.386. (**e**) Array CGH detected gains at chromosome 12p11.1 to 5q12. The log2 ratio was approximately 0.473. (**f**) Array CGH detected gains in chromosome X, one segmental gain of Xp11.21 to Xq11.1 had log2 ratio of 0.674 which is bigger than the other segmental gains of Xp11.22 to Xp11.21 and Xq11.1 to Xq12 with log2 ratio of around 0.473. (**g**) Confirmatory FISH using whole chromosome painting probe for chromosome 5 revealed two normal chromosome 5 in metaphase cells. (**h**) Confirmatory FISH using whole chromosome painting probe for chromosome 5 revealed two normal chromosome 5 plus sSMC der(5)(:p12- > q12.1:) in metaphase cells. (**i**) Confirmatory FISH results of this sSMC using the CEP probe specific for chromosome 12 (green) and the CEP probe specific for chromosome X (red) revealed three green distinct signals and three red distinct signals. (**j**) Confirmatory FISH results of this sSMC using the CEP probe specific for chromosome 12 (green) and the CEP probe specific for chromosome X (red) revealed three green distinct signals and four red distinct signals.

Except for complex sSMCs, multiple sSMCs derived from different chromosomes and presented simultaneously in a cell was another complicated situation, especially when multiple sSMCs occured in a mosaic form. In the present study, case P19 (Fig. 2) had 2–4 sSMCs in different cells based on chromosome analysis (Fig. 2a,b,c). To identify the component of all the markers, aCGH was performed and detected gains in three chromosomes, including chromosome 5, 12, and X. For the gain from chromosome X, one segmental gain of Xp11.21 to Xq11.1 had log2 ratio of 0.674 which is bigger than the other segmental gains with log2 ratio of around 0.473 (Fig. 2f). For the gains of chromosome 5p12 to 5q12.1 and chromosome 12p11.1 to 12q12, log2 ratio were evenly around 0.386 and 0.473, respectively (Fig. 2d,e), indicating that the sSMC derived from chromosome 5 was in mosaic form. These aCGH findings suggested that sSMC(12) was presented in all cells, whereas sSMC(5) was not, and the remaining two sSMCs might derivative from chromosome X. The small derivative X chromosome was more likely originated from segment of Xp11.21 to Xq11.1, and the large derivative X chromosome was more likely originated from segment of Xp11.22 to Xq12. Based on the results of aCGH, FISH probes including whole chromosome 5 painting probes, chromosome 12 and X centromere probes were applied for confirmatory study. The FISH results confirmed aCGH findings and indicated that all cells had marker chromosome der(X)(:p11.22- > q12:) and der(12)(:p11.1- > q12:), whereas the marker chromosome der(X)(:p11.21- > q11.1:) and der(5)(:p12- > q12.1:) were not observed in all cells (Fig. 2g,h,i,j). Therefore, for these complicated cases such as complex sSMCs and multiple sSMCs, the aCGH results provided a clear picture of the components of the sSMCs in one test demonstrating the advantages for sSMCs characterization. Patients with multiple sSMC were rarely reported in the literature. In the current study, the baby girl P19 had four sSMCs derived from chromosome 5, 12, and X, respectively. She showed centriculap septal defect, trileaflet aortic valve, left aortic arch, unusual origin of right coronary artery, almost interrupted aortic arch, and small transverse arch at birth, and heart was too small to be medical sized. Though review the sSMC database^20^, two cases with min(12)(:p11.1 → q12:) are clinical normal indicate that this sSMC is more likely to be benign, whereas the cases with similar origin of sSMC (5) and (X) present multiple abnormalities including developmental delay, facial dysmorphisms, small hand and foot, and many more. Thus, the other three sSMC derived from chromosome 5 and X are more likely responsible for the phenotype of this baby girl.

Array CGH may detect the underlying submicroscopic deletions and duplications missed by G-banding. In one of the Emanuel syndrome (supernumerary der(22)t(11;22) syndrome) cases (P17), array CGH uncovered an additional chromosome abnormality, an extra gain of ~637 Kb located at chromosome 20p12.2. Although the clinical significance of this gained region is not clear at this moment, it gave us a hint that some sSMC patients had additional chromosomal anomalies, and they could have been missed if aCGH was not performed. In some cases, the additional chromosomal anomalies, beside the sSMC, may also contribute to the phenotypes^22^. When the sSMC was detected, we cannot assume that it was the sSMC responsible for the patient’s phenotype, especially when no clear genotype-phenotype correlation had been made. To avoid the misdiagnosis, advanced techniques including array CGH were needed to result in more data for those sSMC carriers and clarify the genotype-phenotype correlation for future clinical service.

**Figure 3:**
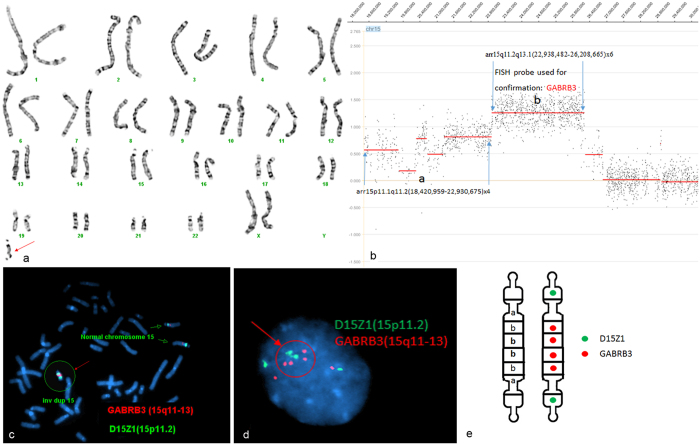
Cytogenetic and molecular results of case P5. The marker is highlighted by a red arrow in a, c, and d. (**a**) G-banding revealed a karyotype 47,XX, +mar in all studied cells. (**b**) The sSMC of case P6 characterized after aCGH as partial tetraploid: arr15p11.1q11.2(18,420,959–22,812,656)x4 and partial hexaploid: arr15q11.2-q12(22,818,696–26,863,401)x6. (**c**) Confirmatory FISH result of this sSMC revealed two distinct hybridization signals of D15Z1 and one large GABRB3 signal on the marker chromosome in metaphase cells. (**d**) Confirmatory FISH result of this sSMC revealed six distinct hybridization signals of GABRA3 and four distinct hybridization signals of D15Z1 in one interphase cell. (**e**) Schematic of sSMC. The structure of the sSMC was der(15)(pter- > q13.1::q13.1- > q11.2::q11.2- > q13.1::q13.1- > pter).

In some cases, the chromosome segments duplicated more than once and then formed the sSMC. Array CGH showed the copy number changes due to the sSMCs in such a case. In case P6 (Fig. 3), aCGH showed segments gain in chromosome 15 with different gain ratio. The segmental gain of 15p11.1 to 15q11.2 (“a” in Fig. 3b) had gain ratio from 0.165 to 0.798 indicating that this region was more likely duplicated twice and formed a inv dup shape sSMC. The different gain ratio in this region may be due to the fact that this region has many copy number variants. The segmental gain of 15q11.2 to 15q13.1 (“b” in Fig. 3b) had gain ratio of 1.242 indicating the copy number of this segment was greater than four. To characterize the exact copy number of segment “b” as well as confirm array findings, FISH utilizing the D15Z1 probe located at 15p11.2 and the GABRB3 probe located at 15q11 to 15q13 was performed. Subsequent FISH testing revealed two distinct hybridization signals of D15Z1 and one large GABRB3 signal on the marker chromosome in metaphase cells (Fig. 3c). The large signal was formed by four GABRB3 signals close to each other, which can be seen clearly in the interphase cells (Fig. 3d). Thus, the combination of aCGH and FISH results clearly demonstrated that the structure of the sSMC was der(15)(pter- > q13.1::q13.1- > q11.2::q11.2- > q13.1::q13.1- > pter) (Fig. 3e). In this sSMC, a segment involving the PWS/AS critical region was a partial hexasomy. This marker is very unique with only 7 similar cases previously reported^23^. In this study, five cases carried sSMC originated from chromosome 15 containing the PWS/AS critical region (15q11q13) (P2, P3, P4, P5, P6). The duplication of chromosome 15q11q13 had been well characterized and might be associate with developmental delay, mental retardation, ataxia, seizures, and behavioral problems^23, 24^. The PWS/AS critical region was dosage sensitive. Patients with more copies of this region may develop more sever phenotype than patients who had less copies^23^.

Conveniently, aCGH identifies all of the gene components of the sSMC and other underlying chromosomal anomalies in one test. The information provided by aCGH is very useful for both clinical physicians and basic medical science researchers. First, it provides important information for clinical services if the sSMC contains a known dosage-sensitive gene based on aCGH findings. Secondly, if the sSMC is correlated with normal features, the characterization of the sSMC could provide important information about genes that did not lead to abnormalities in the presence of gene dosage imbalances^5, 25^. This phenomenon is called genetic silencing, which may play an important role in the clinical consequences of sSMC^25^. Thirdly, for the sSMC carrier associated with varying clinical features, detailed characterization of the sSMC could provide useful data to establish the phenotype-genotype correlation, and then assist in interpreting the potential phenotypic impact^5, 7, 9^. Finally, aCGH characterization of sSMCs in individuals with special clinical features may contribute to the search for candidate genes or a novel locus associated with unique syndromes^26, 27^.

**Figure 4:**
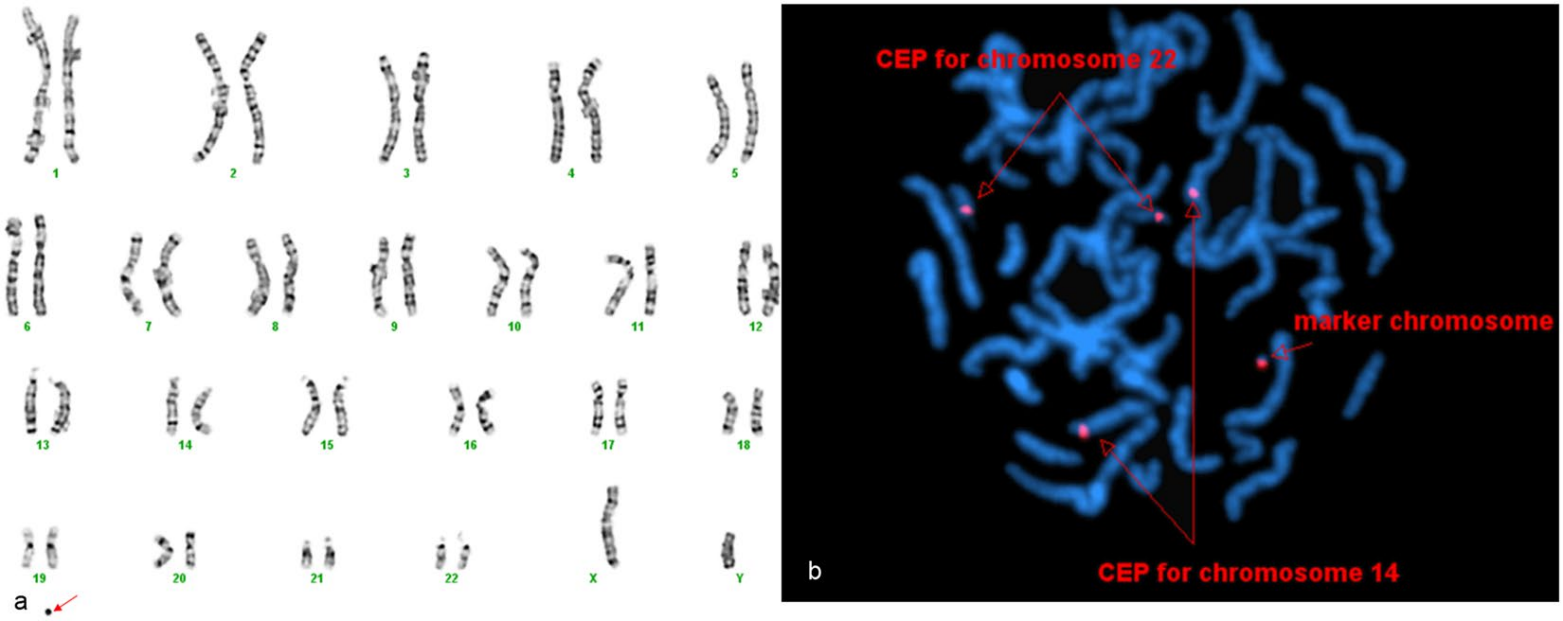
Cytogenetic results of case P13. The marker is highlighted by a red arrow in a and b. (**a**) In twenty-seven out of the thirty studied cells, G-banding revealed a karyotype 47,XY, +mar. In the other three cells, G-banding revealed a normal karyotype 46,XY. (**b**) FISH analysis using the probe specific for centromere 14/22 revealed five distinct signals.

In this study, three cases (P12, P13, P20) were failed to detect the chromosomes origin by aCGH. For case P12, the child inherited the sSMC from his mother who was phenotypically normal. FISH analysis indicated that this sSMC was derived from chromosome 14 or 22. For case P20, the sSMC was firstly identified in the fetus and then was found in her mother who was phenotypically normal. For these two cases, due to no anomalies had been observed, the most possible reason of a negative aCGH result might be that the marker only contained heterochromatin. For case P13, the patients had speech problem, short stature, and failure to thrive at the time when the test was performed. Routine cytogenetics analysis indicated that 90% cells had a very small sSMC (Fig. 4a). FISH analysis using probe cen 14/22 showed signal on the marker chromosome (Fig. 4b). This sSMC was very small and appeared as a dot on karyotype. It may be undetected by NimbleGen 385 K chip if the chip did not have enough probes mapping the marker. The other possibility was that the phenotype was not caused by the sSMC but some undetected reasons else. Due to the limited amount of specimen, no further study was performed.

Although aCGH has many advantages for sSMCs characterization, it cannot take over the banding cytogenetics and FISH. More than half of sSMCs carriers present with mosaicism^3, 5, 28^ and low level mosaicism (<20%) can be missed by aCGH. Additionally, if the sSMC contains only heterochromatin, it may not be identified by aCGH. In this study, the failure to detection the chromosomal origin of cases P12 and P20 is more likely due to this reason because the person who carry sSMCs are phenotypically normal. On the other hand, aCGH and FISH complement each other and that aCGH cannot be correctly interpreted without additional cytogenetic and FISH studies. For example, in case 19, it takes all three methods to clarify the sSMCs characterization.

## Conclusion

This study analyzed twenty sSMC cases using G-banded karyotyping, aCGH, and FISH. The genotype-phenotype correlation of sSMC-related is hard to be established due to the fact that phenotype is determined by the mosaic form, molecular component, and shape of the marker chromosome. In order to establish a stronger for clinical service in the future and avoid miss characterization, more sSMC cases were needed to be detailed characterized. This will help to clarify the variable clinical characteristics of sSMCs and provide additional information to aid clinical service and future research.

## Methods

### Cases studied

From 2000 to 2014, a total of twenty-seven pre and postnatal specimen samples were diagnosed as sSMC carriers at the Genetics Laboratory of the University of Oklahoma Health Sciences Center. Of these twenty-seven samples, due to the limited amount of specimen, seven of the sSMC cases could not be studied further. The remaining twenty cases, nineteen from peripheral blood and one from amniotic fluid samples, were included in this study. This study previously received IRB approval from Institutional Review Board for the Protection of Human Subjects of the University of Oklahoma (IRB# 6299). Informed consent was obtained from all subjects. All experiments in this study, including banding cytogenetics, Oligonucleotide aCGH, and FISH, were performed in accordance with relevant guidelines and regulations.

### Banding cytogenetics

Cultures of peripheral blood and amniotic fluid patient samples were established and harvested according to our standard laboratory protocols. Chromosome preparations were treated with trypsin and stained with Giemsa (G-banded).

### Oligonucleotide aCGH

Genomic DNA was extracted from each patient’s culture cell pellet or from peripheral white blood cells according to our standard operating using the phenol and chloroform method or Nucleic Acid Isolation System (QuickGene-610L, FUJIFILM Corporation, Tokyo, Japan). Two aCGH platforms were used in this study including NimbleGen and Agilent. For NimbleGen aCGH platform, human reference genomic DNA was purchased from Promega (Promega Corporation, Madison, WI, USA). The patient’s DNA and the purchased reference DNA were labeled with either cyanine 3 (Cy-3) or cyanine 5 (Cy-5) by random priming (Trilink Biotechnologies, San Diego, CA, USA). These samples were subsequently hybridized to a NimbleGen high-capacity 385 K, 3 × 720 K or 3 × 1.4 M oligo microarray chip (Roche/NimbleGen System Inc., Madison, WI, USA) by incubating in a MAUI Hybridization System (BioMicro Systems, Salt Lake City, UT, USA) for 18–40 hours according to NimbleGen’s CGH protocols. The array was scanned at 532 nm and 635 nm using the NimbleGen MS 200 Microarray Scanner (NimbleGen System Inc, Madison, WI, USA). NimbleScan and SignalMap (NimbleGen System Inc, Madison, WI, USA) were applied for data analysis. For Roche 385 K and 720 K chip, the genomic positions were determined using GRCh36/hg18, UCSC Genome Browser. For Roche 1.4 M chip, the genomic positions were determined using GRCh37/hg19, UCSC Genome Browser. For Agilent aCGH platform, human reference genomic DNA was purchased from Agilent (Agilent Corporation, Santa Clara, CA, USA). The patient’s DNA and the purchased reference DNA were labeled with either cyanine 3 (Cy-3) or cyanine 5 (Cy-5) by random priming (Agilent Corporation, Santa Clara, CA, USA). Patient DNA (labeled with Cy-3) was combined with a normal control DNA sample (labeled with Cy-5) of the same sex and hybridized to a Agilent 2 × 400 K oligo microarray chip (Agilent Technologies, Santa Clara, CA, USA) by incubating in Agilent’s Microarray Hybridization Ovens (Agilent Technologies, Santa Clara, CA, USA). After a 40 hours hybridization at 67 °C, the slides were washed and scanned using the NimbleGen MS 200 Microarray Scanner (NimbleGen System Inc, Madison, WI, USA). Agilent’s CytoGenomics 2.7 software (Agilent Technologies, Santa Clara, CA, USA) were applied for data analysis. The genomic positions were determined using GRCh37/hg19, UCSC Genome Browser.

### FISH

Prior to 2008, FISH analysis was performed to detect the chromosome origin of sSMCs on four cases (case# P4, P6, P8, P13). In 2008, aCGH was performed on these same four cases to characterize the details of each sSMC. In the remaining sixteen cases, aCGH was first applied to characterize the marker and then FISH analysis was performed on unique or complex cases to verify the results of the aCGH. The selection of probes was based on the gain region detected by aCGH. The majority of probes used in this study were commercial probes, including chromosome 13/21, 14/22, and X centromere probes, the TEL probe located at 12p13, the D15Z1 probe located at 15p11.2, the SNRPN probe specific for the PWS/AS critical region, the GABRB3 probe located at 15q11-13, the PML probe located at 15q22, the TelVysion 16p probe specific for 16pter, the TUPLE1 probe located at 22q11.2, the ASRA probe located at 22q13, and whole chromosome 5 and 22 painting probes (WCP)(Abbott Molecular, Des Plaines, IL). For other confirmatory FISH, we applied homemade probes including the RP11-362K14 probe specific for 3q26 and the RP11-496N6 probe specific for 11qter. The clone DNA was labeled using a random priming reaction, and the labeled DNA was precipitated and subsequently used as probes for standard FISH assays. All homemade FISH probes were validated internally prior to the confirmatory study.

### Ethics approval and consent to participate

This study previously received institution IRB approval. IRB#: 6299.

## References

[1] Liehr T, Claussen U, Starke H (2004). Small supernumerary marker chromosomes (sSMC) in humans. Cytogenetic and genome research.

[2] Liehr T, Weise A (2007). Frequency of small supernumerary marker chromosomes in prenatal, newborn, developmentally retarded and infertility diagnostics. International journal of molecular medicine.

[3] Malvestiti F (2014). De novo small supernumerary marker chromosomes detected on 143,000 consecutive prenatal diagnoses: chromosomal distribution, frequencies, and characterization combining molecular cytogenetics approaches. Prenatal diagnosis.

[4] Jafari-Ghahfarokhi H (2015). Small supernumerary marker chromosomes and their correlation with specific syndromes. Advanced biomedical research.

[5] Liehr T (2006). Small supernumerary marker chromosomes–progress towards a genotype-phenotype correlation. Cytogenetic and genome research.

[6] Anderlid BM (2001). Detailed characterization of 12 supernumerary ring chromosomes using micro-FISH and search for uniparental disomy. American journal of medical genetics.

[7] Huang B, Solomon S, Thangavelu M, Peters K, Bhatt S (2006). Supernumerary marker chromosomes detected in 100,000 prenatal diagnoses: molecular cytogenetic studies and clinical significance. Prenatal diagnosis.

[8] Graf MD (2006). Redefining the risks of prenatally ascertained supernumerary marker chromosomes: a collaborative study. Journal of medical genetics.

[9] Starke H (2003). Small supernumerary marker chromosomes (SMCs): genotype-phenotype correlation and classification. Human genetics.

[10] Vetro A (2012). Unexpected results in the constitution of small supernumerary marker chromosomes. European journal of medical genetics.

[11] McDermid HE (1986). Characterization of the supernumerary chromosome in cat eye syndrome. Science.

[12] Speicher MR, Gwyn Ballard S, Ward DC (1996). Karyotyping human chromosomes by combinatorial multi-fluor FISH. Nature genetics.

[13] Nietzel A (2001). A new multicolor-FISH approach for the characterization of marker chromosomes: centromere-specific multicolor-FISH (cenM-FISH). Human genetics.

[14] Pietrzak J (2007). Molecular cytogenetic characterization of eight small supernumerary marker chromosomes originating from chromosomes 2, 4, 8, 18, and 21 in three patients. Journal of applied genetics.

[15] Tsuchiya KD (2008). Unexpected structural complexity of supernumerary marker chromosomes characterized by microarray comparative genomic hybridization. Molecular cytogenetics.

[16] Trifonov V (2008). Complex rearranged small supernumerary marker chromosomes (sSMC), three new cases; evidence for an underestimated entity?. Molecular cytogenetics.

[17] Wang NJ, Liu D, Parokonny AS, Schanen NC (2004). High-resolution molecular characterization of 15q11-q13 rearrangements by array comparative genomic hybridization (array CGH) with detection of gene dosage. American journal of human genetics.

[18] Jang W (2016). Identification of small marker chromosomes using microarray comparative genomic hybridization and multicolor fluorescent *in situ* hybridization. Molecular cytogenetics.

[19] Olszewska M (2015). Genetic dosage and position effect of small supernumerary marker chromosome (sSMC) in human sperm nuclei in infertile male patient. Sci Rep.

[20] Liehr T. Small supernumerary marker chromosomes. http://ssmc-tl.com/sSMC.html [accessed 05/15/2017] (2017).

[21] Sun M (2014). 16p13.3 duplication associated with non-syndromic pierre robin sequence with incomplete penetrance. Molecular cytogenetics.

[22] Nelle H (2010). Presence of harmless small supernumerary marker chromosomes hampers molecular genetic diagnosis: a case report. Molecular medicine reports.

[23] Kraoua L (2011). Hexasomy of the Prader-Willi/Angelman critical region, including the OCA2 gene, in a patient with pigmentary dysplasia: case report. European journal of medical genetics.

[24] Burnside RD (2011). Microdeletion/microduplication of proximal 15q11.2 between BP1 and BP2: a susceptibility region for neurological dysfunction including developmental and language delay. Hum Genet.

[25] Stephane P, Genevieve L (1999). Prenatal supernumerary r(16) chromosome characterized by multiprobe FISH with normal pregnancy outcome. Prenatal diagnosis.

[26] Faucz FR (2011). Mosaic partial trisomy 19p12-q13.11 due to a small supernumerary marker chromosome: a locus associated with Asperger syndrome?. American journal of medical genetics. Part A.

[27] Zerem A (2011). Mosaic marker chromosome 16 resulting in 16q11.2-q12.1 gain in a child with intellectual disability, microcephaly, and cerebellar cortical dysplasia. American journal of medical genetics. Part A.

[28] Liehr T (2010). Somatic mosaicism in cases with small supernumerary marker chromosomes. Current genomics.

